# Pathophysiologic Consequences of Early Coarctation Stenting in a Longitudinal Porcine Model

**DOI:** 10.1002/ccd.70692

**Published:** 2026-06-21

**Authors:** Leah M. Gober, Michael A. Stellon, Matt A. Culver, Amy Frenkel, Dana Irrer, Colleen M. Witzenburg, Alejandro Roldán‐Alzate, Luke Lamers

**Affiliations:** ^1^ Surgery University of Wisconsin‐Madison Madison Wisconsin USA; ^2^ Biomedical Engineering University of Wisconsin‐Madison Madison Wisconsin USA; ^3^ School of Medicine and Public Health University of Wisconsin‐Madison Madison Wisconsin USA; ^4^ Pediatrics, Division of Cardiology University of Wisconsin‐Madison Madison Wisconsin USA; ^5^ Radiology University of Wisconsin‐Madison Madison Wisconsin USA; ^6^ Mechanical Engineering University of Wisconsin‐Madison Madison Wisconsin USA

**Keywords:** coarctation, hemodynamics, longitudinal model, Renata Minima

## Abstract

**Background:**

Novel stent technologies offer early intravascular treatment options for coarctation (COA). Mid‐to‐long‐term cardiovascular responses to early COA stent interventions are undefined.

**Aims:**

We studied a porcine model of COA, with and without early stent implantation with serial dilation to adult aortic diameters, aiming to define longitudinal cardiovascular changes.

**Methods:**

In total, 18 swine were studied: 6 shams, 6 COA controls (CC), and 6 COA stents (CS). Surgical COA was induced at 2 weeks (4.5 kg) followed by stent implant at 6 weeks, with serial dilations at 12 and 20 weeks (80 kg). All animals were terminally studied at 20 weeks.

**Results:**

Successful stent implantation with serial dilation matching somatic growth occurred in all CS animals with predictable stent performance and elimination of COA. No in‐stent stenosis observed. Final stent dimensions equaled the proximal descending aorta (DAo) of shams (16.8 ± 1.5 vs. 17.5 ± 0.8, *p* = 0.3); however, DAo narrowing (13.3 ± 1.1 mm) just distal to the stent without gradient was created because of stent shortening, and this DAo segment demonstrated decreased vessel wall collagen content. Four of 6 animals developed peri‐stent aneurysms treated with covered stents. CS prevented post stenotic dilation and collateralization and resulted in near normalization of catheter‐derived hemodynamics. Cardiac MRI documented similar ventricular function, cardiac index, and myocardial tissue characterization between groups. Early COA stenting was associated with increased systemic arterial elastin content.

**Conclusion:**

In this swine COA model, early stent implants effectively dilated to adult aortic dimensions, prevented blood pressure gradients, collateralization, and adverse myocardial remodeling. Early stenting with serial dilations altered systemic arterial elastin content, and additional stents may be necessary to enlarge the adjacent DAo. Aneurysm formation frequently occurred.

**Abbreviations**
CC: coarctation controls
COA: coarctation
CS: coarctation stents
DAo: descending aorta
HTN: hypertension
LFA: left femoral artery
LV: left ventricle
post‐COA: post‐coarctation of the aorta
pre‐COA: pre‐coarctation of the aorta
RCA: right carotid artery

## 1 Introduction

Coarctation of the aorta (COA) is a commonly encountered congenital heart disease (CHD), occurring in 1 in 2500 live births, with management strategies that vary depending on the anatomy of the lesion, coexisting conditions, and timing of presentation [1]. High‐grade stenosis and complex COA typically require intervention early in life. Less severe forms of COA present in a delayed fashion, often detected as a result of early‐onset hypertension (HTN) or heart murmur [2]. Unfortunately, successful relief of COA does not ensure normal cardiovascular health [3]. Adults with repaired COA frequently demonstrate structural and functional changes in the systemic vasculature leading to a high prevalence of HTN [3, 4, 5]. The extent of vascular changes and the risk of HTN are greater in individuals repaired at a later age [5, 6]. Defining the evolution of vascular changes secondary to COA and the consequences of early intervention on these changes will lead to more effective treatment strategies.

Endovascular interventions for COA have been performed in both adults and children, with multi‐center trials demonstrating favorable long‐term outcomes [7, 8, 9]. A major limitation to COA endovascular intervention has been the lack of physiologically ideal, growth‐accounting, and low‐profile stents [10]. Novel stents recently received FDA approval for the treatment of COA in infants [11]. Early results are promising [11, 12]; however, detailed studies are needed to confirm efficacy over time, and animal models may highlight important pathophysiologic outcomes that cannot be obtained through human study [13, 14, 15]. With this knowledge, we aim to report results following early stent implantation and serial dilation in a rapidly growing animal model and thereby better define the mid‐term cardiovascular response to early COA stent interventions.

## 2 Materials and Methods

### 2.1 Animal Model and Surgical COA Creation

**Figure 1:**
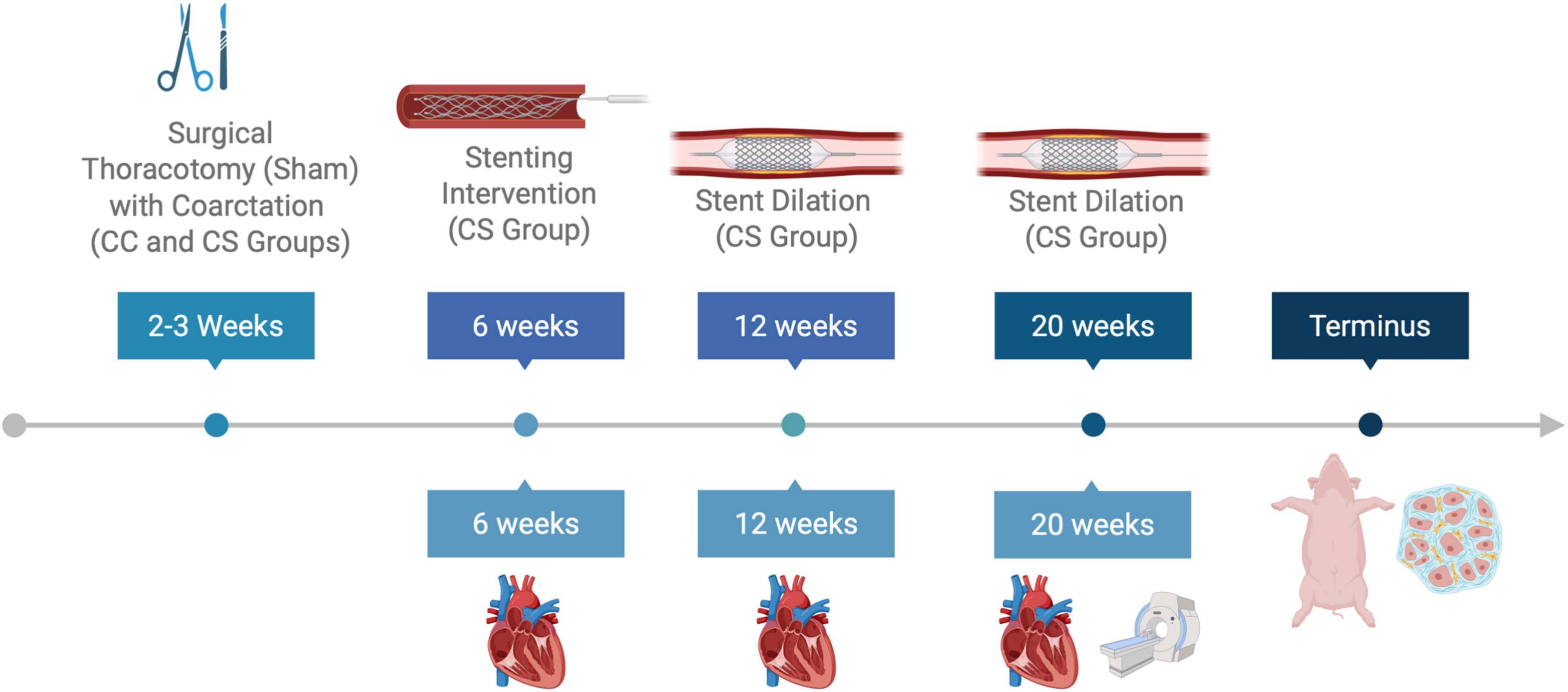
Experimental timeline.

Eighteen male domestic swine were obtained from the University of Wisconsin Swine Research and Teaching Center, and a discrete COA was surgically created at 2 weeks of age (4.5 ± 1 kg) in 12 animals. At 6 weeks of age (9 ± 2 kg), the coarctation stent (CS) group (*n* = 6) underwent COA stent implantation with subsequent dilation at 12 and 20 weeks. All animals were terminally studied at 20 weeks. The Institutional Animal Care and Use Committee of the University of Wisconsin reviewed and approved this study, and the overall experimental timeline is shown in Figure 1.

Surgical COA was created as previously published by our group [16]. Briefly, through a left thoracotomy, the proximal descending aorta (DAo) was isolated and circumferentially mobilized. At this point, the sham group (*n* = 6) procedure was considered complete. For the coarctation control (CC, *n* = 6) and CS group (*n* = 6), the aorta was cross‐clamped, and a 1 cm longitudinal incision was made in the left lateral wall to induce intimal trauma. The arteriotomy was closed, plicated, and wrapped with an 8 mm diameter GORE‐TEX tube graft to ensure aortic narrowing with somatic growth.

### 2.2 Stent Intervention Sequence

The Renata Minima (Renata Medical Company, Costa Mesa, CA) stent was used in the CS group and had a 100% implant success rate with a single, experienced operator (L.L.). This stent is designed for implantation through 4 Fr vascular access with capacity for serial dilation to adult blood vessel diameters [11]. Stent deployment utilized the 4‐Fr delivery system with an 8 mm balloon (14 ± 2 atm) to achieve the desired implant diameter. Two animals required post‐deployment dilation for complete apposition using low‐pressure balloons (10–11 mm Tyshak II – Braun Interventional Systems, Bethlehem, PA). CS animals did not receive anti‐platelet therapy through the study timeframe. Serial stent dilations occurred with semi‐compliant (Evercross – Medtronic, Minneapolis, MN) and non‐compliant (Atlas – Becton Dickinson, Franklin Lakes, NJ) balloons at 2 mm diameter increments at the 12‐ and 20‐week time points with the goal to dilate the stent to diameters equal to the DAo at the diaphragm. Pressures and angiograms were recorded before and after each intervention.

### 2.3 Twenty‐Week Cardiac Catheterization

**Figure 2:**
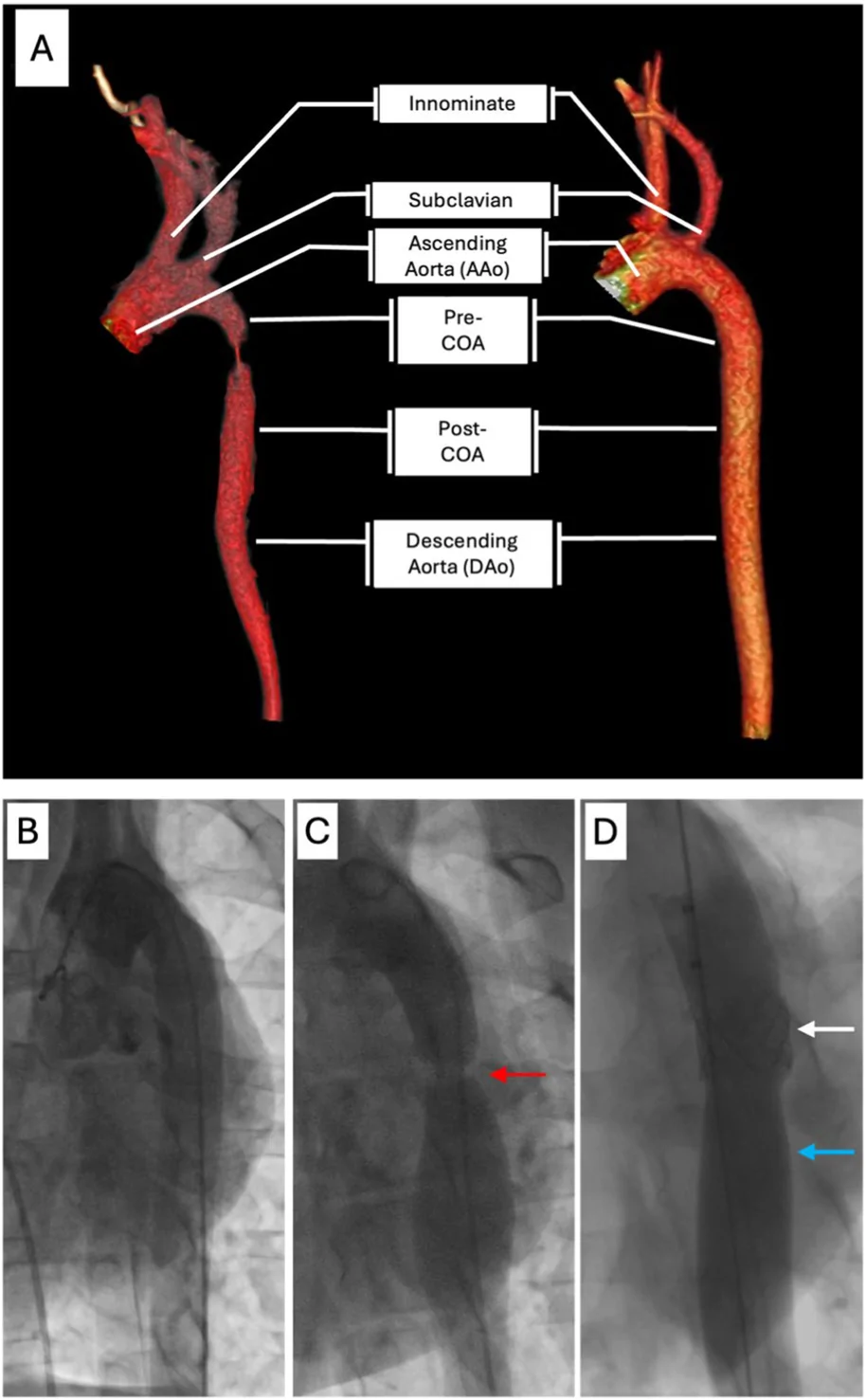
Anatomic locations for aortic measurements. (A) Representative locations from MRA at which measurements were obtained. Angiographic image of sham (B), CC (C) with COA noted (red arrow), and CS subject (D) with stent (white arrow) matching adjacent aortic diameter (blue arrow).

Following induction (telazol IM, 6 mg/kg and xylazine IM, 2 mg/kg) and general anesthesia (isoflurane 2%–5%), femoral artery access was obtained percutaneously, and intravenous heparin was given. Heart rate (HR) and beat‐by‐beat hemodynamics were obtained in the left ventricle (LV), ascending aorta (AAo), and DAo using fluid‐filled catheters attached to a pressure transducer at rest and following dobutamine stress challenge (5 mcg/kg/min infusion). Single plane angiography with maximum systolic aortic dimensions was recorded at four locations: (1) proximal DAo, distal to the L subclavian artery (pre‐COA), (2) site of surgical COA (COA), (3) distal to COA to assess post stenotic dilation (post‐COA), and (4) DAo at the diaphragm (Figure 2).

### 2.4 Twenty‐Week Noninvasive Cardiac Magnetic Resonance

MRI was performed under general anesthesia prior to the 20‐week catheterization on a 3 T imaging system (Discovery MR750, GE Healthcare, Waukesha, WI) using gadobenate dimeglumine contrast [17]. A balanced steady state free precision (bSSFP) sequence was used for conventional cardiac imaging. Short‐axis cine images were segmented to determine variables of LV anatomy and function including mass, wall thickness, end‐systolic and diastolic volumes, stroke volumes, ejection fraction (EF), and cardiac output (CO). Modified look‐locker inversion recovery (MOLLI) sequence‐generated parametric T1 maps at index short‐axis planes were used to calculate native and post contrast global LV myocardial T1 values and extracellular volume.

**Figure 3:**
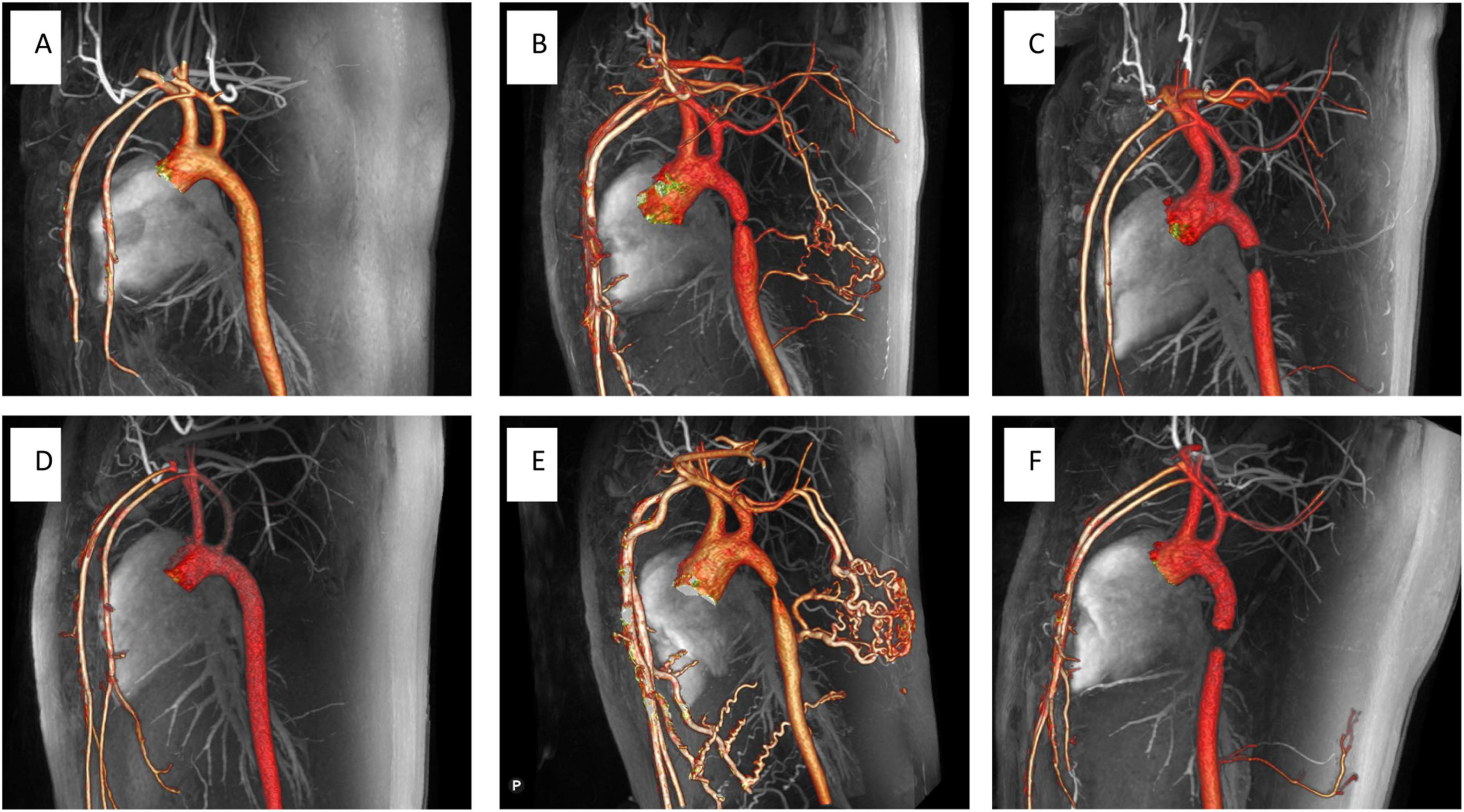
MRA representation of collateralization. Lack of collateralization from the internal mammary arteries in the sham (A, D) and CS group (C, F) compared to extensive collateralization in the CC group (B, E).

Through‐plane velocity‐encoded 2D phase contrast (2D PC) CINE images and angiographic measures were obtained at seven double oblique planes perpendicular to the direction of the flow in the AAo, innominate artery, left subclavian artery, pre‐COA, COA, post‐COA, and DAo at the diaphragm (Figure 2A). bSSFT, MOLLI, and PC images were segmented, after which diameters and flow quantification were performed using dedicated cardiovascular analysis software (CVI42, Circle Cardiovascular Imaging Inc., Calgary, Canada). Maximum and minimum artery areas on the ECG‐gated PC CINE magnitude images were used to calculate relative area change (RAC = Amax − Amin/Amax). A qualitative assessment, focusing on collateralization, was performed using a maximum intensity projection (MIP) from the magnetic resonance angiography (MRA) images (Figure 3).

### 2.5 Histology

After final procedures, all animals were euthanized with IV potassium chloride. Histologic analysis was performed on cross‐sections of the right carotid artery (RCA) and the left femoral artery (LFA) following our published approach, which was utilized previously on tissue from the pre‐ and post‐COA aorta [18]. Adjacent sections, 5 µm in thickness, were stained with either Masson's trichrome (MTC) and Verhoeff–Van Gieson (VVG) to visualize collagen and elastin, respectively. A Nikon Eclipse NiU microscope at a 10× objective was used to obtain whole slide images of each stained section. Segmentation using Adobe Photoshop was performed independently by researchers, blinded to sample group, on VVG‐stained samples to quantify the luminal area and the combined area of the media and intima. These measures were normalized to animal weight. Segmentations were also mapped onto images of neighboring MTC‐stained slices such that a custom MATLAB code developed by Bersi et al. [19] isolated the medial collagen and elastin area fractions (AFs) via colorimetric analysis.

### 2.6 Statistics

Continuous data are presented as mean ± standard deviation, and categorical data are presented as fractions and percentages. Shapiro−Wilk's test was used to confirm the normal distribution of the data. One‐way and two‐way ANOVA were used where appropriate with post hoc multiple comparisons tests. Intergroup variability or change was analyzed with a paired *t*‐test. A *p*‐value of < 0.05 was considered statistically significant. Statistical analysis was performed using GraphPad Prism 10 (GraphPad Software LLC, Boston, MA).

## 3 Results

### 3.1 Animal Growth

Animal weights at the time of the 20‐week terminal procedures were not significantly different between groups and consistent with adult‐sized pigs (Table 1) [20]. For this reason, the only values standardized to body surface area are done so in clinical interpretation.

**Table 1:** Twenty‐week catheterization derived hemodynamic metrics.

	Sham	Sham, +Dob	CC	CC, +Dob	CS	CS, +Dob	CS, post‐dilation
Weight (kg)	80 ± 18		86 ± 14		83 ± 12		
LV, SBP^†^	111 ± 19	162 ± 29	123 ± 36	195 ± 40	118 ± 20	179 ± 25	
AAo, SBP^†^	107 ± 16	146 ± 11	126 ± 33	175 ± 32	113 ± 17	163 ± 20	105 ± 13
DAo, SBP	107 ± 16	147 ± 13	102 ± 24	125 ± 22	104 ± 18	149 ± 29	105 ± 15
LV, DBP	7 ± 4	12 ± 5	9 ± 4	14 ± 5	11 ± 6	14 ± 5	
AAo, DBP	81 ± 12	99 ± 8	89 ± 23	120 ± 23	79 ± 15	111 ± 32	82 ± 17
DAo, DBP	79 ± 12	94 ± 9	86 ± 21	107 ± 22	79 ± 16	112 ± 22	80 ± 17
AAo, MAP	90 ± 12	114 ± 6	101 ± 27	138 ± 25	91 ± 16	128 ± 27	89 ± 15
DAo, MAP	88 ± 13	111 ± 8	91 ± 23	113 ± 22	87 ± 17	124 ± 23	88 ± 16
AAo, PP^a^	26 ± 6	47 ± 15	37 ± 13	55 ± 15	34 ± 4	53 ± 19	23 ± 8
DAo, PP^a^^,^^†^	27 ± 6	52 ± 13	16 ± 5	18 ± 7	26 ± 7	38 ± 14	25 ± 8
Gradient, SBP^a^^,^ b ^,^ c ^,^^†,§^	0 ± 2	−2 ± 9	24 ± 11	50 ± 19	9 ± 5	14 ± 20	1 ± 2
Gradient, DBP	2 ± 1	5 ± 1	3 ± 3	13 ± 8	1 ± 4	−1 ± 13	−2 ± 1
HR (bpm)	89.5 ± 11.6	130.3 ± 18.2	87.5 ± 13.8	150.8 ± 22.5	107.7 ± 15.4	147 ± 23.7	

Abbreviations: AAo, ascending aorta; bpm, beats per minute; DAo, descending aorta; DBP, diastolic blood pressure; Dob, dobutamine; HR, heart rate; LV, left ventricle; MAP, mean arterial pressure; PP, pulse pressure; SBP, systolic blood pressure.

^a^ Varies significantly between sham and CC, *p* < 0.04; †varies significantly between sham (+Dob) and CC (+Dob), *p* < 0.04.

^b^ Varies significantly between sham and CS, *p* < 0.04.

^c^ Varies significantly between CC and CS, *p* < 0.001; ^§^varies significantly between CC (+Dob) and CS (+Dob), *p* < 0.005.

### 3.2 Serial Stent Intervention Outcomes

Successful stent implantation occurred in all CS animals at 6 weeks with relief of COA narrowing and peak systolic pressure gradients. With the 6‐week stent implant, the minimum COA diameter increased from 4.7 ± 0.7 to 7.8 ± 0.9 mm (*p* < 0.05) and stents were re‐dilated to match somatic growth of the adjacent aorta at 12 weeks (7.8 ± 0.2 to 10.4 ± 1.1 mm) and 20 weeks (10.4 ± 1.4 to 16.8 ± 1.5 mm). At each time point, pressure gradients significantly improved post‐dilation (Supporting Information S5: Table 1). Predictable stent shortening occurred from 17 mm at implant to 13.3 ± 1.1 mm at 20 weeks [11]. No in‐stent stenosis occurred at any time point.

At the 12‐week procedure, two animals had diminutive right femoral arteries on ultrasound that could not be accessed suggesting femoral artery injury with initial stent implant. Four of six animals developed peri‐stent aneurysms (Supporting Information S1: Figure 1). One aneurysm developed between the 6‐ and 12‐week intervention, two aneurysms developed between the 12‐ and 20‐week intervention, and the final aneurysm/contained dissection occurred with 20‐week serial stent dilations. In two animals, aneurysms were treated with covered stents (NuDEL CP, Braun Interventional Systems Inc, PA, USA) as would be standard of care in clinical practice.

### 3.3 Twenty‐Week—Angiography

Pre‐COA and DAo dimensions at the diaphragm were similar between groups. For the CC group, the COA narrowed to 5.5 ± 1.7 mm with associated post‐COA aortic dilation (Table 2). The final CS COA dimensions equaled the proximal DAo of shams (CS 16.8 ± 1.5 mm vs. sham 17.5 ± 0.8 mm, *p* = 0.3); however, the native aorta just distal to the stent in the CS group measured only 13.3 ± 1 mm (Figure 2D).

**Table 2:** Twenty‐week angiographic dimensions.

	Sham	CC	CS, pre‐dilation	CS, post‐dilation
Pre‐COA	18.6 ± 1.3	16.4 ± 2.6	17.4 ± 1.6	
COA^a^^,^ b ^,^ c	18.5 ± 1.7	5.5 ± 1.7	10.4 ± 1.4	16.8 ± 1.5
Post‐COA^a^^,^ c	18.8 ± 1.7	23.6 ± 5.4	18.6 ± 1.4	
Diaphragm	17.7 ± 1.9	18.3 ± 4.7	16.7 ± 2.2	

*Note:* Measurements represent aortic diameters (mm).

Abbreviation: COA, coarctation.

^a^ Varies significantly between sham and CC, *p* < 0.01.

^b^ Varies significantly between sham and CS, *p* < 0.001.

^c^ Varies significantly between CC and CS, *p* < 0.01.

### 3.4 Twenty‐Week—Hemodynamics

Systolic blood pressures (SBPs) in the LV and AAo trended higher in the CC group (Table 1); however, there was no statistical difference between groups in baseline pressures in any location (LV, AAo, and DAo). The CC group had an SBP gradient (AAo:DAo, 24 ± 11 mmHg) that was significantly greater than the sham (0 ± 2 mmHg) and CS group pre (9 ± 5 mmHg) and post 20‐week (1 ± 2 mmHg) stent dilation. Pulse pressure (PP) was increased in the AAo of the CC group compared to shams (37 ± 13 vs. 26 ± 6 mmHg, *p* = 0.03) and trended towards significance when comparing CS to shams (34 ± 4 vs. 26 ± 6 mmHg, *p* = 0.15). As expected, DAo PP for the CC group was diminished.

SBP response to dobutamine was more pronounced, but not significantly so, in the LV and AAo for the CC and CS groups. The COA gradient increased to 50 ± 19 mmHg in the CC group, compared to 14 ± 20 and −2 ± 9 mmHg in the CS and sham group (Table 1). AAo PP differences seen at baseline did not persist; however, DAo PP was greatest in shams (52 ± 13 mmHg), intermediate in the CS group (38 ± 14 mmHg), and remained significantly blunted in the CC group (18 ± 7 mmHg). HR at baseline and in response to dobutamine was similar between groups.

### 3.5 Cardiac MR—LV Assessment and Tissue Characterization

MRI analysis of ventricular function (Table 3) yielded no statistically significant differences among groups; however, there was a trend towards higher LV ESV with lower SV and EF in the CC and CS groups. Cardiac index was similar between groups. LV mass and peak wall thickness were greatest in the CC group without reaching statistical significance. T1 mapping of myocardial tissue characterization was similar between groups.

**Table 3:** Twenty‐week cardiac MR tissue characterization and left ventricular volumetric analysis.

	Sham	CC	CS
LV EDV (mL)	124 ± 31	129 ± 22	123 ± 22
LV EDV (mL)/TBSA	82 ± 12	82 ± 8	82 ± 10
LV ESV (mL)	47 ± 13	55 ± 18	53 ± 8
LV ESV (mL)/TBSA	31 ± 8	34 ± 9	36 ± 5
SV (mL)	78 ± 25	75 ± 13	70 ± 19
LV mass (g)	98 ± 25	111 ± 19	101 ± 7
LV mass (g)/TBSA	64 ± 9	70 ± 8	68 ± 7
EF (%)	62 ± 7	58 ± 10	56 ± 8
CI (L/min/m^2^)	5.5 ± 1	4.8 ± 1	5.4 ± 1
CO (L/min)	9 ± 3	8 ± 1	8 ± 2
Peak wall thickness (ms)	14 ± 1	17 ± 2	15 ± 2
Native T1 (ms)	1146 ± 68.2	1060 ± 61.6	1105 ± 82.3
ECV (%)	32.7 ± 8.5	34.7 ± 6.4	28.0 ± 10.2

*Note:* LV volumes: two‐way ANOVA, Tukey's multiple comparisons, *p* < 0.05. LV mass, EF, CI, CO, peak wall thickness, T1, and ECV: one‐way ANOVA, Tukey's multiple comparisons, *p* < 0.05. No statistically significant differences among groups. *N* = 6/6/6.

Abbreviations: CI, cardiac index; CO, cardiac output; ECV, extracellular volume; EDV, end diastolic volume; EF, ejection fraction; ESV, end‐systolic volume; g, grams; LV, left ventricle; m, meters; mL, milliliters; ms, milliseconds; SV, stroke volume; TBSA, total body surface area.

### 3.6 Magnetic Resonance Angiography

Cardiac MRA was used to define additional dimensional and anatomic characteristics of the aortic arch, arch vessels, and collateralization (Figure 3). MRA demonstrated significantly larger ascending aortic diameters in the CC group (Supporting Information S6: Table 2) with trends towards larger innominate and left subclavian arteries compared to CS and shams. CS animals were anatomically similar to shams for all MRA‐derived dimensions. Similar to catheter angiographic aortic diameters, the untreated COA location measured 6.3 ± 1.9 mm. Post‐COA dilation and extensive AAo to DAo collateral formation seen only in the CC group.

### 3.7 Relative Area Change and Net Flows

RAC, a surrogate for vascular compliance, was similar between groups for most measured arterial segments. Only the post‐COA aorta demonstrated increased RAC in the CS group compared to shams (Table 4). Net flow, a composite of the forward and retrograde flow during a cardiac cycle, was calculated within predefined arterial segments and was similar in the AAo and DAo at the diaphragm for all groups. In the CC group, pre‐COA and post‐COA net flows were significantly lower, while innominate and left subclavian artery net flows were increased (Table 4). To determine the flow contribution of collateralization, we calculated the pre‐COA minus DAo flow at the diaphragm. This revealed a higher flow differential in CC animals (−13.3 ± 15.2 mL/cycle), consistent with 34% of flow to the DAo at the diaphragm via collateral circulation. CS net flows were similar to shams for all measured segments.

**Table 4:** Twenty‐week cardiac MR dynamic metrics.

		Sham	CC	CS
Relative area change (%)	AAo	26.3 ± 7.3	27.3 ± 7.2	29.7 ± 5.2
	Pre‐COA	13.2 ± 4.7	12.7 ± 2.5	17.9 ± 6.0
	Post‐COA^b^	10.9 ± 4.1	11.6 ± 11.9	21.1 ± 4.7
	Diaphragm	13.4 ± 8.9	9.0 ± 6.5	14.3 ± 3.9
	Innominate	23.1 ± 1.4	19.5 ± 6.3	27.4 ± 4.2
	Subclavian	11.7 ± 14.6 (*n* = 3)	22.7 ± 7.6	26.7 ± 10.2
Blood flow (mL/cycle)	Pre‐COA diaphragm^a^	8.8 ± 10.5	−13.3 ± 15.2	−0.2 ± 4.9
	AAo	62.7 ± 13	70.5 ± 12.3	66.9 ± 17.3
	Pre‐COA^a^^,^ c	45.7 ± 10.6	26.3 ± 15.5	46.9 ± 9.5
	Post‐COA^c^	37.1 ± 7.7	24.6 ± 12.7	49.6 ± 13.9
	Diaphragm	36.9 ± 9.6	39.6 ± 6.2	47.1 ± 10.8
	Innominate^a^^,^ c	15.7 ± 5.7	30.5 ± 6.1	14.7 ± 3.9
	Subclavian	9.6 ± 2.7 (*n* = 3)	18.1 ± 6.2	5.8 ± 2.9

*Note: N* = 6/6/6 unless otherwise noted. Two‐way ANOVA, Tukey's multiple comparisons, *p* < 0.05.

^a^ Varies significantly between sham and CC, *p* < 0.05.

^b^ Varies significantly between sham and CS groups, *p* < 0.05.

^c^ Varies significantly between CC and CS, *p* < 0.01.

### 3.8 Histology

**Figure 4:**
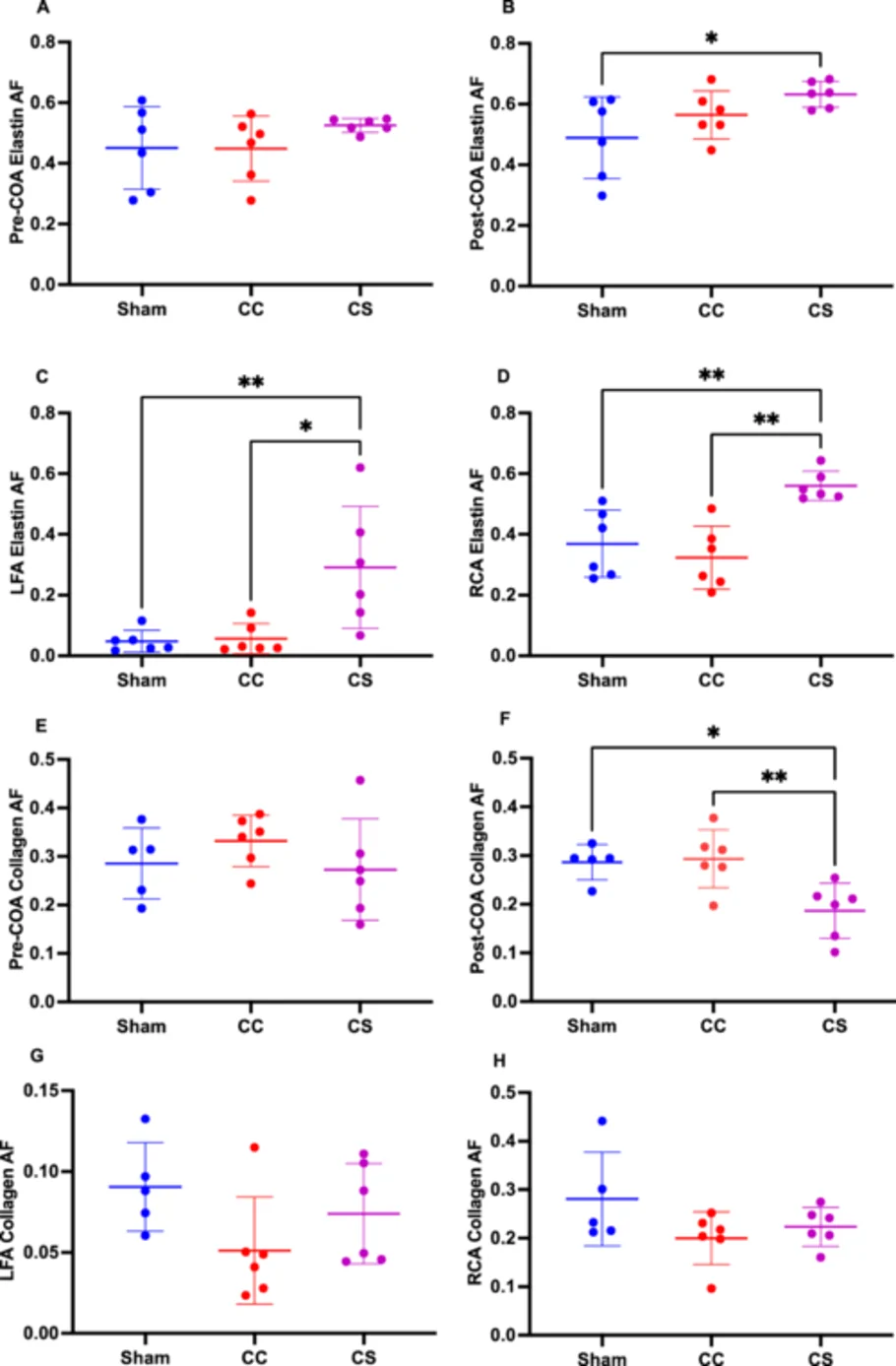
Elastin and collagen area fractions among four histologically evaluated locations: pre‐COA, post‐COA, LFA, and RCA. (A) – (H) are elastin and collagen area fractions for arterial segments labled on the y axis of each graph. The elastin area fraction was higher in the CS group for the post‐COA aorta (*p* = 0.04), the LFA (*p* = 0.009), and the RCA (*p* = 0.007), when compared to shams. However, the collagen content was only significantly different in the post‐COA aorta (*p* = 0.02 vs. shams, *p* = 0.009 vs. CC).

The elastin and collagen AFs determined from colorimetric analysis of four vascular segments (pre‐COA, post‐COA, LFA, and RCA) are shown in Figure 4. The elastin AF was significantly higher in the CS group for all locations (*p* < 0.01) except the pre‐COA aorta, where the CS group still had a trend towards increased elastin content (Figure 4A–D). In contrast, the collagen AF was similar for all measured segments with the exception of the post‐COA aorta in the CS group, where collagen content was decreased. As a quality check, we compared sham elastin and collagen AFs from the four vessel segments (Supporting Information S2: Figure 2), both were significantly lower in the LFA than all other locations, consistent with previously published reports [21]. Of note, the CS group increased elastin fibers appeared to have normal deposition, without breakage or lamellar disruption (Supporting Information S3: Figure 3).

The cross‐sectional tissue area (media + intima) demonstrated pre‐COA wall thinning in the CS group compared to the sham group (*p* = 0.03, Supporting Information S4: Figure 4A). Additionally, there was no pre‐COA wall thickening for the untreated CC group. The LFA was thinner in the CC group compared to the sham group (*p* = 0.05, Supporting Information S4: Figure 4C). Post‐COA aorta and RCA arterial wall areas were equivalent among groups (Supporting Information S4: Figure 4B,D) [18].

## 4 Discussion

There has long been interest in transcatheter therapies for neonatal or early infant management of COA, and novel stent technologies are expanding the potential for minimally invasive therapies early in life. Findings from our neonatal porcine model add to the scant data defining the local and systemic vascular response to early COA stenting. This study demonstrates that early stent implants perform as intended in a swine COA model, capable of alleviating stenosis, dilatable to adult aortic dimensions with predictable shortening while maintaining stent integrity [11, 12]. Early COA stenting prevented collateralization and limited adverse LV remodeling while nearly normalizing the hemodynamic and flow alterations seen in untreated COA. A particularly interesting and not previously defined systemic response to early stenting appears to be global alterations in arterial elastin content associated with arterial wall thinning that may equate to more compliant vessels far from the COA stent. The stimulus for and consequences of this systemic arterial remodeling seen with this swine model of COA in response to early aortic stenting form the basis for future investigation.

This swine model mimics isolated COA in humans, mild to moderate stenosis early in life that is progressive over time and clinically tolerated with preserved LV function. At 6 weeks, with initial stent implantation, the COA measured 4.8 mm with a 10–15 mmHg gradient. This degree of aortic narrowing persisted in untreated controls with a 20‐week COA diameter measuring 5.5 mm, which was associated with a 20–25 mmHg resting gradient that increased to > 50 mmHg with pharmacologic stress. This degree of COA in an 80 kg human with extensive collateralization equates to moderate aortic narrowing and warrants intervention [22]. Surprisingly, this degree of COA was not associated with extensive adverse vascular remodeling (intimal/medial thickening) proximal to the untreated COA as seen with prior animal studies [23, 24, 25]. We attribute this to our unique COA model that it is created in neonatal piglets, and in this rapidly growing animal model, we believe there was only a short time in the animal's life with moderate to severe COA to stimulate adverse remodeling. In comparison, most prior COA animal studies created aortic narrowing in fully‐grown animals [26, 27, 28, 29]. Notably, a “window of aortic plasticity” was observed in the only other animal model to our knowledge in which COA was induced prior to adulthood (at 10 weeks of age in rabbits) [30]. Combining these two animal studies implies the importance of early COA detection and treatment to potentially limit adverse remodeling. Left untreated for longer durations, we suspect the untreated COA animals would develop adverse vascular and cardiac remodeling commonly seen in older patients with untreated COA [31]. Collateralization was largely not observed in the adult models and appears to proceed pre‐COA arterial thickening and adverse remodeling suggesting an alternative vascular response in early life [26, 27, 28].

Technical aspects of early stenting for treatment of this COA model included experiencing two femoral artery occlusions following stent implant; however, we did not use anticoagulation nor perform post procedure arterial access imaging as would be standard of care following early COA stenting in clinical practice. Stent shortening with serial dilation resulted in narrowing of the adjacent “normal” aorta without an associated BP gradient. For the entire aorta to be dilatable to adult diameters, one may need to place additional stents to compensate for this phenomenon. In this swine COA model, aneurysm formation occurred in 4/6 animals with serial dilation. The surgical technique to create COA, three aortic interventions over a short period of time (14 weeks), and the high inflation pressures necessary to achieve desired implant diameters (14 ± 2 atm) all likely contributed to aneurysm formation, yet vascular injury and aneurysm formation are known sequela and long‐term complications following both endovascular and surgical COA interventions [32, 33]. Aneurysms could be effectively excluded by covered stenting at the 20‐week catheterization when the femoral arterial vessels were of adequate size to accommodate the vascular access necessary for large‐diameter covered stent placement.

Interestingly, arterial elastin content adjacent to the COA stent and more so distant from the region of intervention (femoral and carotid arteries) increased following early COA stenting with serial dilations, and aortic collagen content immediately distal to the stented segment was decreased. Neither of these two histological findings has been previously described in response to aortic stenting or following COA surgical repair. This swine COA model, created at 2 weeks of age, occurs within the timeframe of continued arterial elastin deposition in neonatal piglets [34]. Prior animal and human studies demonstrate sharp rises in aortic elastin content in the first 2–3 months of life, then arterial elastogenesis is thought to cease [35, 36, 37, 38]. Arterial elastin enhances vessel capacitance and over time undergoes gradual degradation creating arterial stiffness thought to contribute to HTN with aging [39]. Abundant data exists surrounding compensatory mechanism of amplified elastin deposition in pediatric lung pathologies resulting in altered lung compliance, yet less is known about systemic arterial elastin deposition in response to localized vascular interventions [40, 41, 42]. Bendeck and Langille observed higher elastin content in the RCAs of 6‐week‐old rabbits with left external carotid artery ligation, and this difference was not present following similar arterial ligation in adult animals [37]. Similarly, Matsuda et al. reported a significant increase in aortic elastin content of young rats who underwent extensive exercise between ages 9 and 27 weeks [43]. In these studies, increased arterial elastin content improved arterial compliance implying cardiovascular benefit. With this study, only stented animals demonstrated increased arterial elastin content suggesting early COA stenting stimulates elastogenesis in response to localized stent material, increased localized aortic stiffness, and/or repeat aortic dilations. The increased elastin fibers had a normal histological appearance suggesting beneficial systemic remodeling. COA stent animals also had decreased aortic wall collagen immediately distal to the stented aortic segment. This finding is contrary to localized long‐term response to coronary artery stenting, where increased collagen deposition is part of the later phase of neointimal proliferation and vascular remodeling [39]. The aortic segment with decreased collagen was sampled from the narrowed stretched aorta that occurred in response to stent shortening with serial dilations, and likely increases the risk for aneurysm formation warranting further study and surveillance. These vascular alterations are likely the consequence of early COA stenting and non‐stent type‐specific changes. It is unclear but seems unlikely that similar findings would occur in response to early isolated COA angioplasty or surgery. The clinical consequences of these arterial changes, if confirmed in humans following early COA stenting, need to be considered when choosing COA treatment strategies.

### 4.1 Limitations

The isolated COA created for this study occurs in a normal aorta that lacks intrinsic structural abnormalities known to be present in CHD affecting the aorta like COA with bicuspid aortic valve [22]. This swine COA model was created via a lateral wall incision to create intimal/aortic trauma. We attempted to complete aortic transection with end‐to‐end anastomosis wrapped with an external band to mimic typical COA surgical repair, but neonatal operative mortality was prohibitive, so we settled on this model. The untreated COA was moderate to severe so physiologically effective, but the lateral wall incision COA creation likely contributed to the high incidence of aneurysm formation. The study is also limited by sample size, lack of use of different types of stents for treatment of COA for comparison, and relatively short experimental time course. Twenty‐week animals represent a juvenile swine population. Additionally, we saw significant growth between 12 and 20 weeks. With this knowledge, an ideal study would trace subjects through to full adulthood, with appropriately spaced investigational time points to mirror the growth trajectory.

## 5 Conclusion

In this swine COA model, early stent implants dilated to adult aortic dimensions preventing collateralization and adverse hemodynamic consequences. Additional large diameter stents may be necessary to enlarge adjacent aortic narrowing created because of stent shortening, and covered stent therapy was often required to repair resulting aneurysms. Early COA stenting with serial dilations did alter arterial elastin content distant from localized COA stents and forms the basis for future investigation. These findings highlight anatomic and histologic changes in a swine COA model with known limitations and are not intended to advocate this therapy as the standard of care for the treatment of COA in small children.

## Conflicts of Interest

The authors declare no conflicts of interest.

## Supporting information


**Figure S1**: At 20‐week catheterization, 2 animals with peri‐stent saccular aneurysm formation (A,C, red arrow) treated with covered stent placement (Nudel CP) and aneurysmal exclusion (B,D).

**Figure S2:** Elastin and collagen area fractions of the four arterial sections for all Sham animals demonstrate lower elastin (A) and collagen (B) area fractions in the LFA when compared to all other locations.

**Figure S3:** Elastin fibers appeared to have normal deposition, without breakage or lamellar disruption in CC (A) and CS (B) femoral artery samples.

**Figure S4:** Vessel cross‐sectional tissue area, as measured by the summation of the medial and intimal wall areas and normalized to animal weight, demonstrated pre‐COA wall thinning (A) in the CS group, no difference in the post‐COA (B), LFA wall thinning in the CC group (C) and no difference in the RCA (D). *p< 0.05.

**Table S1:** Serial stent angiographic and blood pressure measurements.

**Table S2:** 20‐week MRA‐derived dimensions.

## Data Availability

The data that support the findings of this study are available from the corresponding author upon reasonable request.
