# Supplementary material for: Pathophysiologic Consequences of Early Coarctation Stenting in a Longitudinal Porcine Model
Source: Catheter Cardiovasc Interv. 2026 Jun 21;108(3):732–42. doi: 10.1002/ccd.70692 (PMC13532476; doi:10.1002/ccd.70692)

Supplemental Figure 1: At 20-week catheterization, 2 animals with peri-stent saccular aneurysm formation (A,C, red arrow) treated with covered stent placement (Nudel CP) and aneurysmal exclusion (B,D).

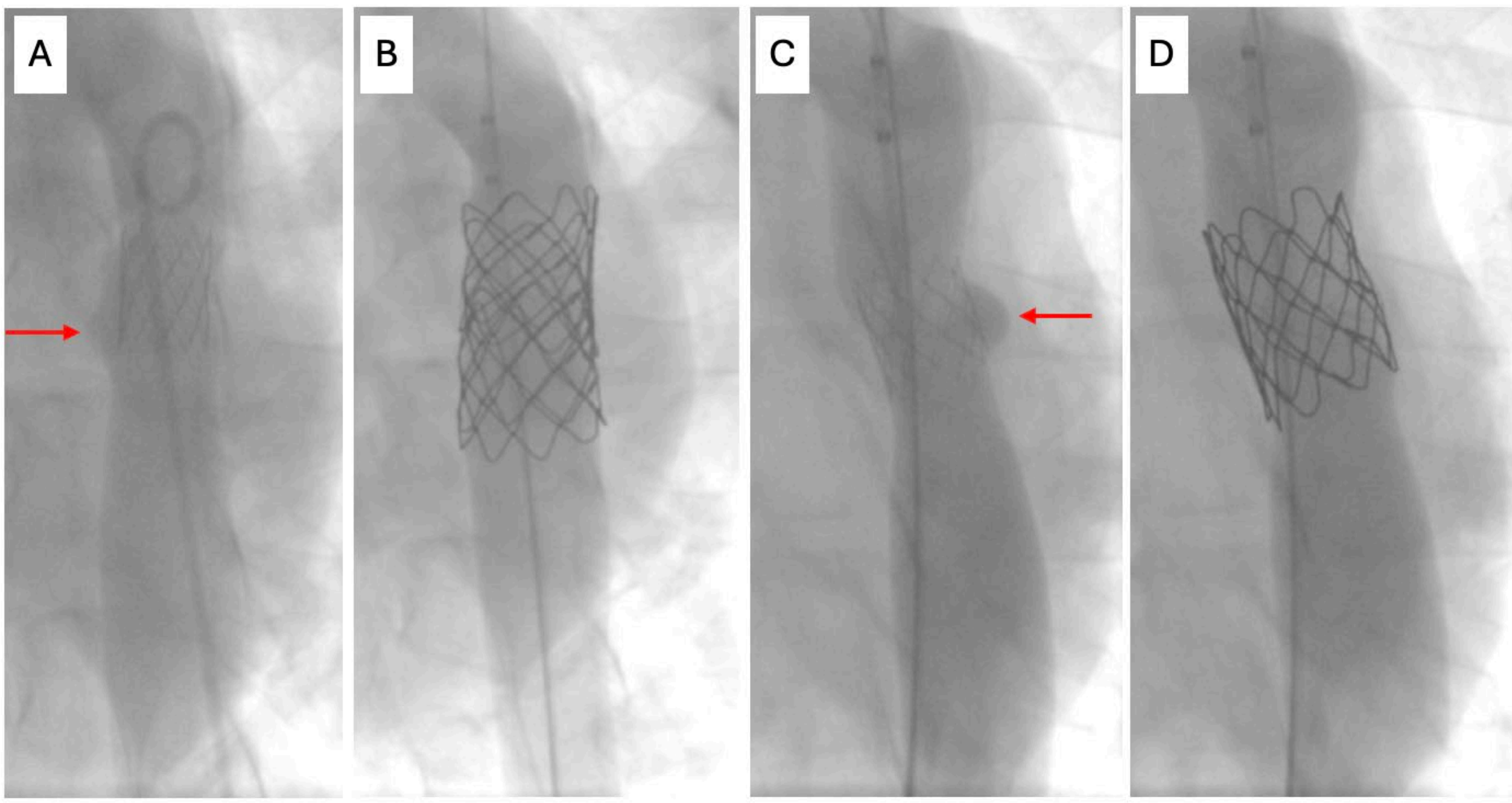

Supplement: Supplementary file 1 [file CCD-108-732-s002.pdf]
