# Supplementary material for: Pathophysiologic Consequences of Early Coarctation Stenting in a Longitudinal Porcine Model
Source: Catheter Cardiovasc Interv. 2026 Jun 21;108(3):732–42. doi: 10.1002/ccd.70692 (PMC13532476; doi:10.1002/ccd.70692)

Supplemental Figure 2: Elastin and collagen area fractions of the four arterial sections for all Sham animals demonstrate lower elastin (A) and collagen (B) area fractions in the LFA when compared to all other locations.

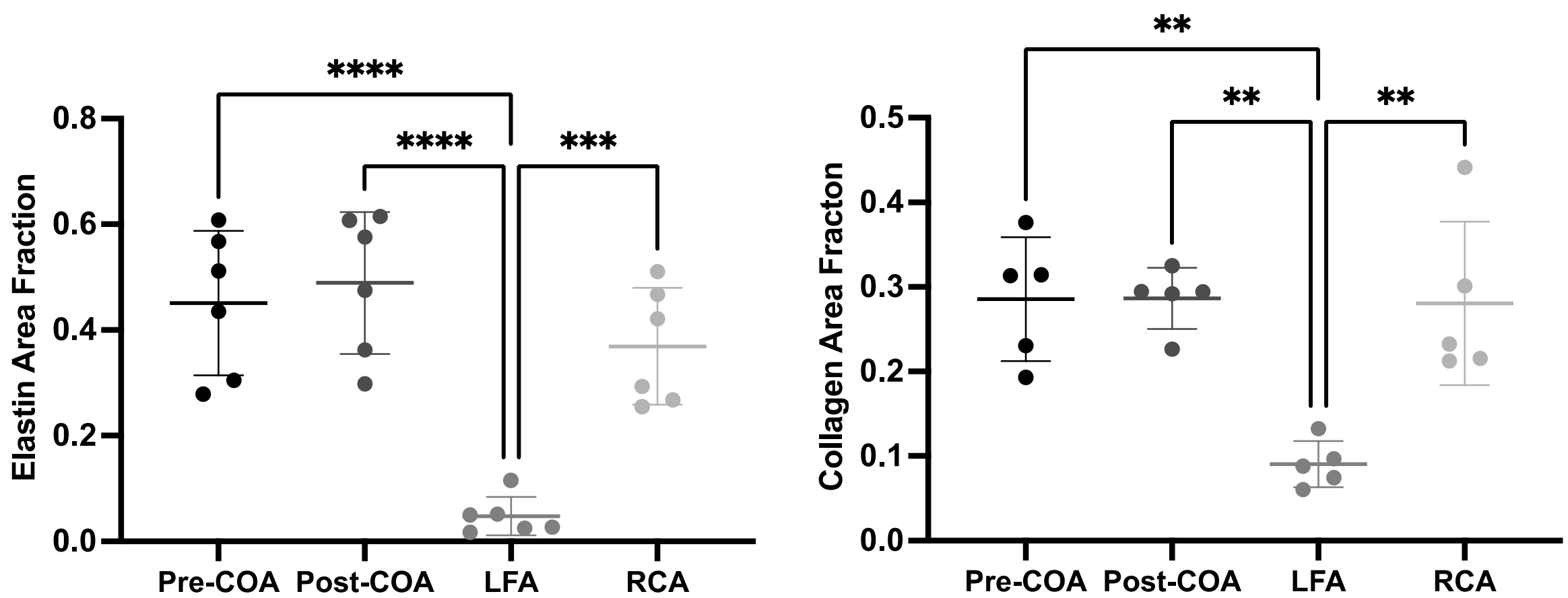

Supplement: Supplementary file 2 [file CCD-108-732-s005.pdf]
