# Supplementary material for: Pathophysiologic Consequences of Early Coarctation Stenting in a Longitudinal Porcine Model
Source: Catheter Cardiovasc Interv. 2026 Jun 21;108(3):732–42. doi: 10.1002/ccd.70692 (PMC13532476; doi:10.1002/ccd.70692)

Supplemental Figure 4: Vessel cross-sectional tissue area, as measured by the summation of the medial and intimal wall areas and normalized to animal weight, demonstrated pre-COA wall thinning (A) in the CS group, no difference in the post-COA (B), LFA wall thinning in the CC group (C) and no difference in the RCA (D). * P< 0.05.

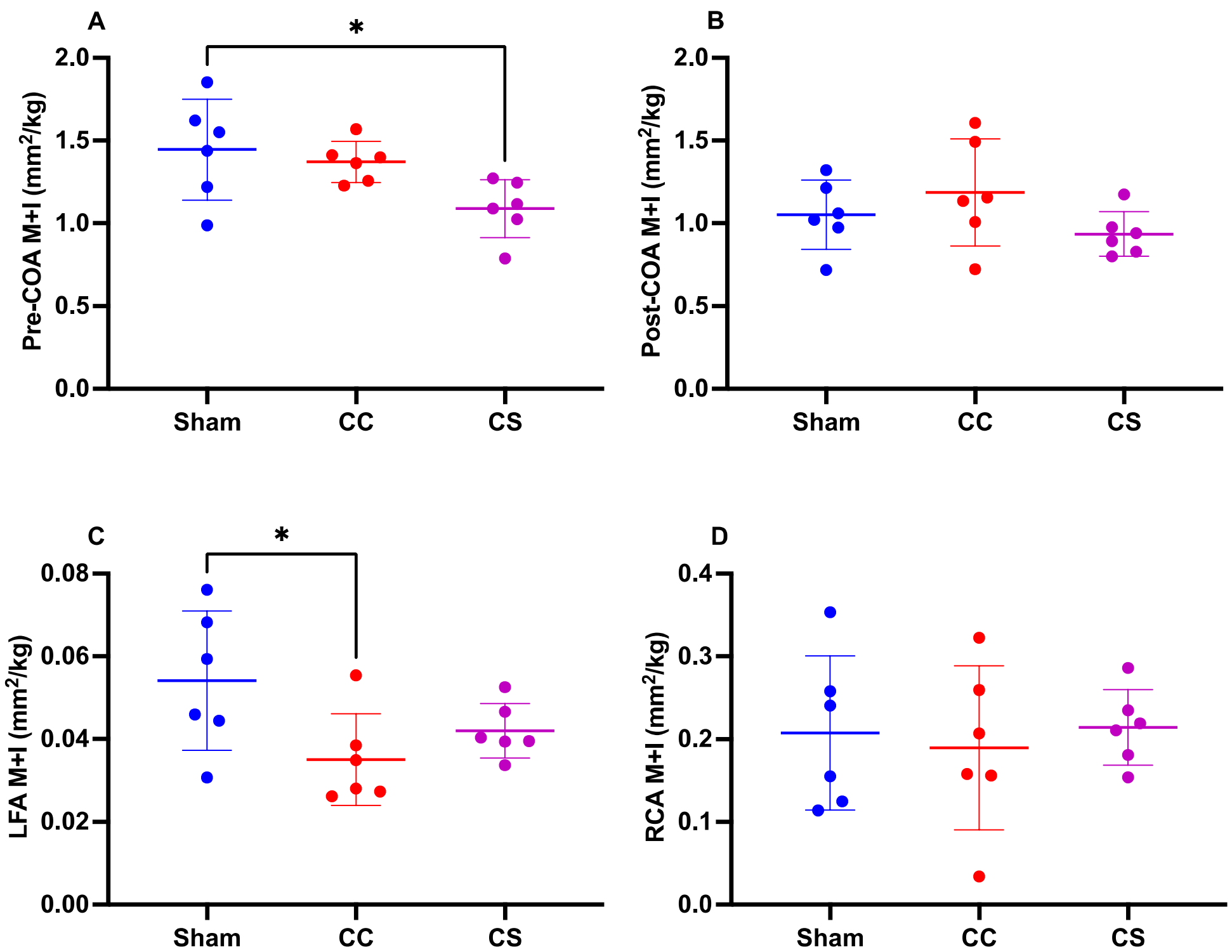

Supplement: Supplementary file 3 [file CCD-108-732-s006.pdf]
