# Supplementary material for: Pathophysiologic Consequences of Early Coarctation Stenting in a Longitudinal Porcine Model
Source: Catheter Cardiovasc Interv. 2026 Jun 21;108(3):732–42. doi: 10.1002/ccd.70692 (PMC13532476; doi:10.1002/ccd.70692)
Supplement: Supplementary file 4 [file CCD-108-732-s001.docx]

***Supplemental Figure 3:*** Elastin fibers appeared to have normal deposition, without breakage or lamellar disruption in CC (A) and CS (B) femoral artery samples.
