# Supplementary material for: Pathophysiologic Consequences of Early Coarctation Stenting in a Longitudinal Porcine Model
Source: Catheter Cardiovasc Interv. 2026 Jun 21;108(3):732–42. doi: 10.1002/ccd.70692 (PMC13532476; doi:10.1002/ccd.70692)
Supplement: Supplementary file 5 [file CCD-108-732-s003.docx]

***Supplemental Table 1: Serial stent angiographic and blood pressure measurements.***

|  | **Stent Dilation Time point** | **Diameter (mm)** | **p-value** | **Pressure (SBP)** | **p-value** |
| --- | --- | --- | --- | --- | --- |
| **COA, 6 weeks** | Pre | 4.8±0.78 | 0.0001 | 13.3±6.8 | 0.001 |
|  | *Post* | *7.8±0.9* |  | 0.3±0.8 |  |
| **COA, 12 weeks** | Pre | 7.8±0.6 | 0.0008 | 6.5±4.3 | 0.01 |
|  | *Post* | *10.4±1.2* |  | 0.8±1.2 |  |
| **COA, 20 weeks** | Pre | 10.4±1.4 | 0.0001 | 13.8±7.1 | 0.001 |
|  | *Post* | *16.8±1.5* |  | 0.2±0.4 |  |
| **Final Stent Metrics** | Diameter | 16.8±1.5 |  |  |  |
|  | Foreshortening (L_initial_ 17mm) | 13.3±1.1 |  |  |  |
