# Supplementary material for: Pathophysiologic Consequences of Early Coarctation Stenting in a Longitudinal Porcine Model
Source: Catheter Cardiovasc Interv. 2026 Jun 21;108(3):732–42. doi: 10.1002/ccd.70692 (PMC13532476; doi:10.1002/ccd.70692)
Supplement: Supplementary file 6 [file CCD-108-732-s004.docx]

***Supplemental Table 2: 20-week MRA-derived dimensions.***

|  | **Sham** | **CC** | **CS** |
| --- | --- | --- | --- |
| **AAo^ac^** | 26.5±5.2 | 33.8±3.4 | 29.0±2.3 |
| **Pre-COA** | 17.8±3.7 | 18.8±2.9 | 18.9±0.8 |
| **COA^a^** | 16.4±3.0 | 6.3±1.9 | -- |
| **Post-COA^ac^** | 18 .0±3.4 | 23.4±5.1 | 16.8±1.5 |
| **DAo** | 14.8±3.3 | 14.9±2.4 | 16.7±0.7 |
| **Innominate** | 12.0±2.3 | 15.4±1.7 | 13.7±0.7 |
| **Subclavian** | 9.9±2.2 | 12.9±1.6 | 10.7±0.9 |

^a^ Varies significantly between Sham and CC, p < 0.01. ^b^ Varies significantly between Sham and CS, p < 0.01. ^c^ Varies significantly between CC and CS, p < 0.05. -- Unable to measure COA diameter within stent due to artifact.
